# Glyco‐Nanogels for Modulating *Pseudomonas aeruginosa* Biofilm

**DOI:** 10.1002/marc.202500807

**Published:** 2025-11-29

**Authors:** Sophia Rosencrantz, Jo Sing Julia Tang, Karina Koenig, Sany Chea, Ruben R. Rosencrantz

**Affiliations:** ^1^ Fraunhofer Institute for Applied Polymer Research IAP Life Science and Bioprocesses Potsdam Germany; ^2^ Fraunhofer Cluster of Excellence Immune‐Mediated Diseases CIMD Frankfurt am Main Germany; ^3^ Brandenburg University of Technology (BTU) Cottbus‐Senftenberg Institute of Materials Chemistry Chair For Biofunctional Polymer Materials Senftenberg Germany

**Keywords:** biofilm modulation, glycopolymers, lectins, nanogels, pathoblockers, *Pseudomonas aeruginosa*

## Abstract

*Pseudomonas aeruginosa* forms biofilms that complicate treatment of infections, with lectins LecA and LecB playing crucial roles in this process. This study investigates the inhibitory effect of glycosylated nanogels on lectin binding and biofilm formation. Nanogels presenting melibiose (α‐galactose) and fucose (β‐fucose) effectively reduce LecA and LecB binding, respectively, in competitive inhibition assays against immobilized glycoproteins. Melibiose nanogels are more potent inhibitors than fucose nanogels, as α‐galactose is more strongly bound by LecA than β‐fucose by LecB. Both types of glycogels have a high impact on *P. aeruginosa* biofilm formation. Notably, the timing of glycogel application significantly influences biofilm dynamics; pre‐treatment leads to a 75% reduction in biofilm formation, whereas treatment after biofilm initiation results in a 60% increase in biofilm growth, suggesting that these glycogels can act as both inhibitors and enhancers of biofilm development. The findings highlight the complexity of carbohydrate‐based interactions in biofilm modulation and underscore the necessity for precise dosing and structural optimization in developing effective strategies against infections caused by biofilm‐forming bacteria.

## Graphical abstract

Nanogels presenting α‐galactose and β‐fucose can significantly alter *Pseudomonas aeruginosa* biofilm dynamics through their interaction with lectins. Application timing is crucial: pre‐treatment reduces biofilm, while treatment after biofilm initiation increases biomass. The dual role of these glycogels as inhibitors and enhancers emphasizes the need for careful application timing and formulation.

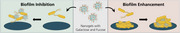

## 1 Introduction

*Pseudomonas aeruginosa* is an opportunistic pathogen rated as critical by the WHO list, indicating that new antibiotics are urgently needed. The bacterium takes advantage of an individual's weakened immune system, causing urinary tract infections, respiratory system infections, dermatitis, soft tissue infections, bacteremia, bone and joint infections, gastrointestinal infections, and a variety of systemic infections. *P. aeruginosa* can be resistant to commonly applied antibiotics; however, general treatment involves the use of cephalosporin antibiotics, like ceftazidime, carbapenems, aminoglycosides, or fluoroquinolones. Most *P. aeruginosa* are resistant to at least one of these drug classes, a few *P. aeruginosa* are even resistant to all of these treatments [1, 2]. Treatment of *P. aeruginosa* is also hampered because of its ability to form biofilms, acting as a protection shield [3]. *P. aeruginosa* forms biofilms as a multifaceted strategy to enhance survival, adapt to environmental challenges, and resist antimicrobial treatments [4, 5, 6].

Biofilm formation is regulated by quorum sensing, a cell‐cell communication mechanism mediated by signaling molecules that can influence the expression of bacterial cell‐surface lectins [7, 8]. Adhesion, binding of virulence factors to cell surfaces, as well as biofilm formation by *P. aeruginosa* are glycan‐mediated interactions. Known virulence factors of *P. aeruginosa* are the lectins LecA (PA‐IL) and LecB (PA‐IIL), which are involved in biofilm build‐up that protects *P. aeruginosa* from immune cells as well as decreases antibiotic susceptibility [9, 10, 11]. Whereas LecA is reported to bind galactose, LecB favors fucosylated structures. It has been proven that glycan‐based drugs are capable of inhibiting biofilm formation by *P. aeruginosa* by being inhibitors for LecA and LecB [12, 13, 14]. Glycan‐based drugs do not act primarily as antibiotics but may be described as pathoblockers [15]. This is a route of circumventing or at least decreasing the fast development of resistance mechanisms of the pathogen, as no systemic stress is caused. The glycan approach is indeed a very different way of dealing with pathogens, as here the metabolism or biological function of the bacteria is not affected.

Several alternatives to classical antibiotics are already being researched or are in early preclinical stages. Besides quorum‐sensing inhibitors or modulators [8, 16, 17] that act glycan‐independently, structures addressing *P. aeruginosa* lectins are coming more and more into focus. Mostly, galactosides or fucosides are oligo‐ or multivalently presented using backbones based on glycopeptide dendrimers [13, 18, 19, 20], calix‐arenes [21, 22, 23], glycoclusters [24, 25, 26, 27, 28, 29, 30] or neoglycoproteins [31, 32]. Lectin binding or inhibition of biofilm formation was reported for these structures, with influence on different linker lengths or multivalences. Potent glycomaterials must comprise a sufficient multivalent mode of ligand presentation. It is known that the affinity of lectins is increased over several orders of magnitude due to the cluster glycoside effect reached by multivalent structures [33]. Also, for certain LecA and LecB inhibitors, it was shown that multivalent ligands are superior compared to monovalent species [25, 26, 34]. Other reports suggested a modified galactose moiety for covalent interaction with LecA to inhibit this lectin as well [35]. An interesting study discussed the use of HMOs as natural glycans that show affinities in the micromolar range [36, 37].

Besides glycan structures to address certain lectins or carbohydrate‐binding receptors, microgels can also be functionalized with lectins, which bind selectively to unique carbohydrate structures on specific pathogens [39]. This high specificity of lectin‐functionalized microgels is an advantage over glycosylated scaffolds, making them particularly valuable in medical applications. Applications range from anti‐biofilm agents that guide antimicrobial nanoparticles to pathogens like *E. coli* and *P. aeruginosa* [39, 40], to “capture‐and‐kill” coatings that bind and then destroy bacteria [41]. They are also explored for therapeutic blood filtering to scavenge pathogens and toxins from the bloodstream [42]. Lectin‐functionalized microgels may face challenges related to the stability of the lectins themselves. In contrast, glycosylated microgels often maintain stability through their inherent chemical properties. In addition, glycosylated microgels are highly versatile and biocompatible, often derived from natural polysaccharides. By mimicking the glycan motifs on cell membranes, these microgels can be engineered to be highly specific for bacterial lectins, such as those on *P. aeruginosa*, effectively scavenging them and preventing the initial stages of infection [43, 44].

Recently, it was reported that glycomimetic orally bioavailable LecB inhibitors are able to minimize the biofilm formation of *P. aeruginosa* in a lab setup [45]. The structures were optimized toward an affinity in the micromolar range and showed good oral bioavailability in mice. Tests were performed with molecular engineered *P. aeruginosa* expressing a red fluorescent protein.

We here developed glycopolymers as polymer‐gel particles presenting melibiose and fucose for addressing LecA and LecB, respectively. The nanogels were characterized in terms of biological function by lectin assays with immobilized glycoproteins and in biofilm tests with *P. aeruginosa*.

## 2 Results and Discussion

### 2.1 Synthesis and Characterization of Nanogels

**SCHEME 1:**
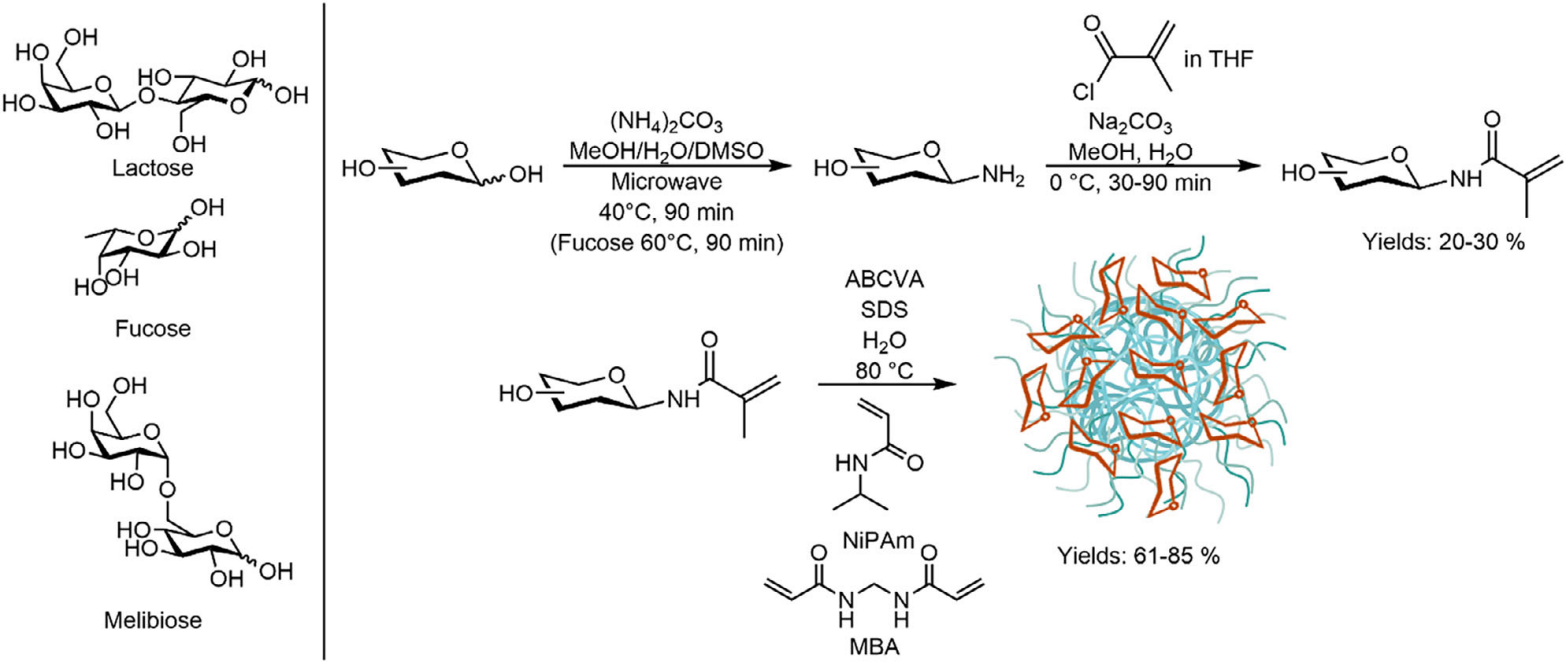
Synthesis scheme for glycogels. Starting from lactose, fucose, or melibiose, glycomonomers were synthesized by microwave‐assisted Kochetkov amination with subsequent reaction with methacryloylchloride. The glycogels were produced via precipitation polymerization with glycomonomer, NiPAm as co‐monomer, and MBA as crosslinker.

For the binding of *P. aeruginosa* lectins, glycosylated nanogels were synthesized via free‐radical precipitation polymerization. The synthesis route led to colloidal polymer networks with multivalent presentation of melibiose and fucose (Scheme 1). Melibiose exhibits a galactose moiety in its α‐configuration, and the pendant fucose groups were functionalized in their β‐configuration, which are known to be recognized by LecA and LecB, respectively. We used a protecting‐group free synthesis approach with high regio‐ and stereo‐selectivity to yield glycomonomers from fucose and melibiose selectively in β‐configuration. The modification takes place specifically at the C1‐position, allowing for lectin recognition. The synthesis consists of only two steps and utilizes cheap raw materials, which makes it an economically feasible process. Although β‐fucose is a non‐optimal ligand for LecB, we investigated whether this drawback could be circumvented by multivalent presentation, keeping the straightforward synthesis approach. For control, NiPAm nanogels without any carbohydrates and lactosylated gels, which carry β‐galactose, were additionally prepared.

The nanogels vary in size, ranging from 50 nm to 1 µm hydrodynamic diameter at 50°C, depending on their containing sugar type (Table 1). Lactose gels revealed the smallest gels, while the fucose gels had the largest diameter. The melibiose gels are half the size of the fucose gels. The sugar content ranges from 0.2 to 0.5 µmol/mg. The fucose and lactose glycogels contain approximately 0.45 µmol/mg sugar, whereas the melibiose gels were measured to have 0.2–0.3 µmol/mg sugar. Thus, the sugar content does not correlate with the gel size.

**TABLE 1:** Yield, sugar content, hydrodynamic diameter, and polydispersity index of nanogels.

Nano‐gel	Yield [%]	Sugar content [µmol/mg]	D_h_(20 ° C) [nm]	PDI [%]	D_h_(50 ° C) [nm]	PDI [%]
G‐I	—	—	460	4.30	218	2.54
MG‐I	61	0.20 ± 0.05	474	31.5	488	20.4
MG‐II	67	0.29 ± 0.06	669	29.7	507	21.2
FG‐I	81.4	0.44 ± 0.06	2928	59.8	975	27.4
FG‐II	85.3	0.47 ± 0.10	1896	84.4	1146	32.7
LG‐I	75.4	0.43 ± 0.03	42.8	56.1	50.3	42.0

G—control gel; MG—melibiose gel; FG—fucose gel; LG—lactose gel.

### 2.2 Inhibition Studies with *Pseudomonas aeruginosa* Lectins

**FIGURE 1:**
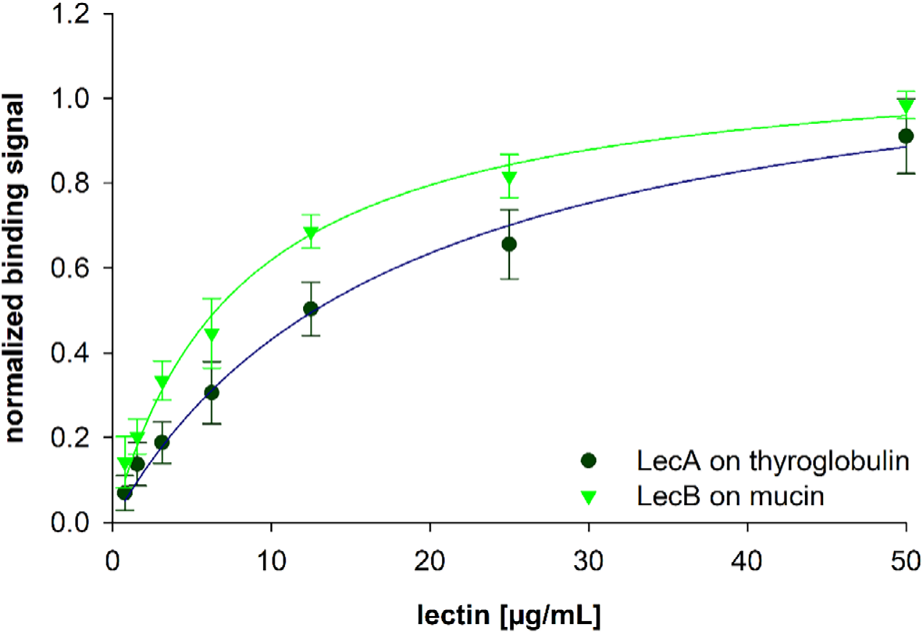
Binding curves of LecA and LecB on immobilized thyroglobulin and mucin, respectively. The immobilized glycoproteins showing successful binding of *P. aeruginosa* lectins are the base for the inhibition assays. The biotinylated lectins were detected with fluorescent streptavidin.

Both *P. aeruginosa* lectins, LecA and LecB, were used for inhibition assays with synthesized glycogels. As immobilized glycoproteins, thyroglobulin and mucin were found to be sufficiently bound by LecA and LecB, respectively, and serve as a base for inhibition studies. Thyroglobulin, therefore, presents galactose moieties while porcine mucin contains fucose residues. The latter we could already prove in binding assays with UEA‐I, another fucose‐binding lectin [46]. Normalized binding curves on these glycoproteins are shown in Figure 1. Regarding absolute values, the binding of LecA on thyroglobulin is much lower than the binding of LecB on mucin.

Two melibiose gels (MG‐I and ‐II), two fucose gels (FG‐I and ‐II), one reference gel without sugar moiety (G‐I), one control gel with lactose (LG‐I), and both free saccharides, melibiose and fucose, were used to inhibit the binding of LecA and LecB in a competitive inhibition assay. Residual binding on the immobilized glycoprotein was determined and compared.

**FIGURE 2:**
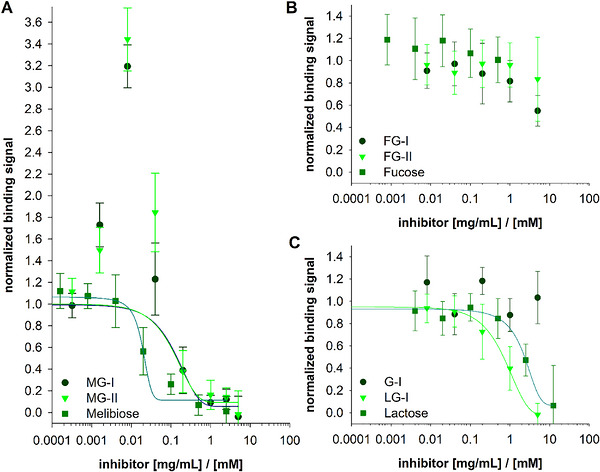
Inhibition of LecA binding to thyroglobulin by glycogels. Melibiose nanogels (MG‐I and MG‐II), as well as free melibiose, inhibit LecA binding (A). Fucose nanogels (FG‐I and FG‐II), as well as free fucose, have no influence on LecA binding (B). Non‐glycosylated control nanogel (G‐I) shows no inhibition, while lactose nanogel (LG‐I), as well as free lactose, are also able to inhibit LecA binding with lower potency (C). Nanogels were applied in mg/mL and free saccharides in mm.

**FIGURE 3:**
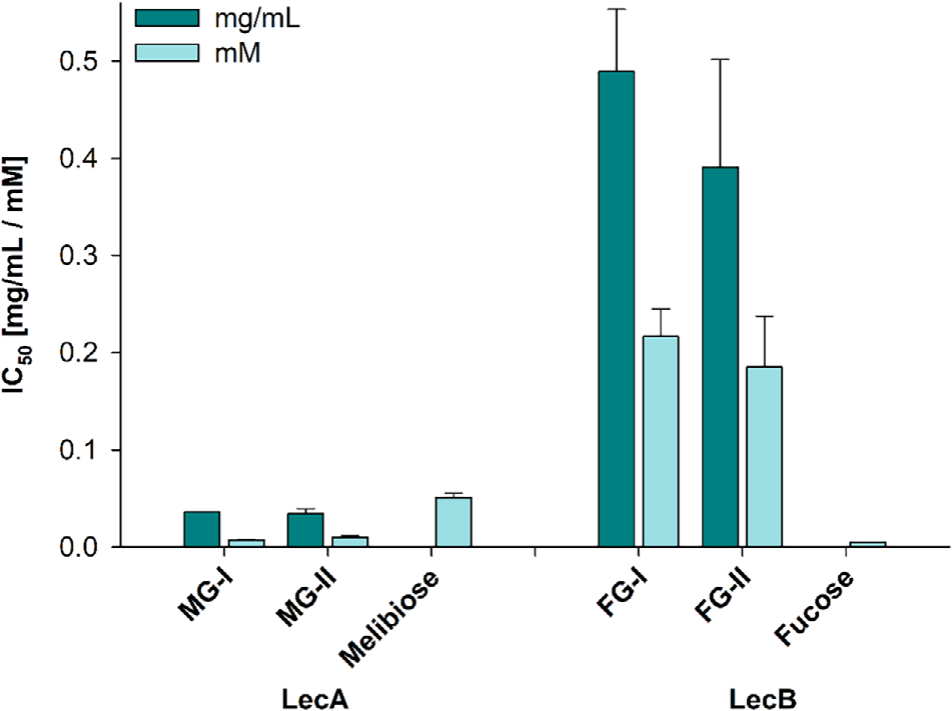
IC_50_ values of melibiose glycogels (MG‐I and MG‐II), as well as free melibiose toward LecA and fucose glycogels (FG‐I and FG‐II), as well as free fucose toward LecB. For the glycogels, the values are given in mg/mL and mm, for the free saccharides in mm.

As expected, simultaneous incubation of melibiose‐presenting gels and LecA on immobilized thyroglobulin resulted in decreasing binding signal with higher gel concentration (Figure 2A). LecA was inhibited by both melibiose gels, with complete inhibition at 1 mg/mL. Here, the small difference between the sugar content in the two gels does not affect the inhibitory potency regarding the calculated IC_50_ value in mg/mL, which is for both nanogels approximately 35 µg/mL (Figure 3). The accessibility of all sugar moieties seems to be limited, and this limit is already reached for both melibiose gels. The IC_50_ value calculated in mm sugar content is therefore slightly lower for MG‐I than MG‐II because of the lower sugar content (Table 1). Interestingly, at low glycogel concentrations between 0.001 and 0.0025 mg/mL, the LecA binding signal was dramatically increased up to 3.5 times the starting signal. LecA, as a tetramer, could be crosslinked via melibiose gels at low concentrations, where not all binding sites are occupied and therefore able to bind the cluster to immobilized thyroglobulin. This is underlined by a significantly lower IC_50_ value calculated in mm for the melibiose gels compared to free melibiose, indicating a multivalent effect. As control, fucose gels as well as free fucose did not act as inhibitors of LecA, confirming its selectivity for galactose (Figure 2B). Not only α‐galactose, present in melibiose, but also β‐galactose seems to be bound by LecA as lactose gel, and free lactose could inhibit LecA binding (Figure 2C). However, the inhibition potency is approximately ten times lower than for α‐galactose. This is in consensus to literature stating that α‐galactose is the natural ligand and β‐galactose the less preferred ligand for LecA [47, 48].

**FIGURE 4:**
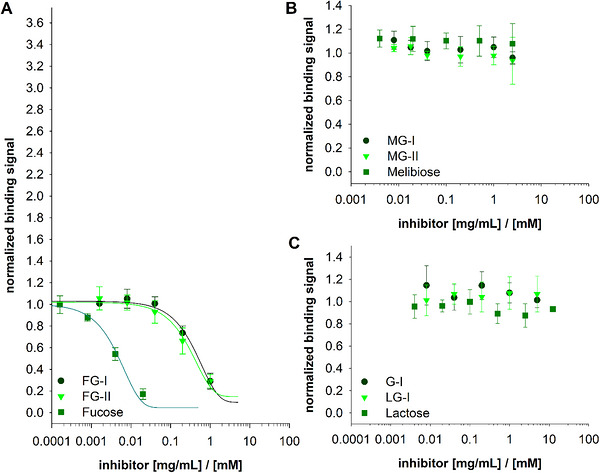
Inhibition of LecB binding to mucin by glycogels. Fucose nanogels (FG‐I and FG‐II), as well as free fucose, inhibit LecB binding (A). Melibiose nanogels (MG‐I and MG‐II), as well as free melibiose, have no influence on LecB binding (B). Non‐glycosylated control nanogel (G‐I) and lactose nanogel (LG‐I), as well as free lactose, also show no inhibition potency (C). Nanogels were applied in mg/mL and free saccharides in mm.

The fucose‐binding LecB was inhibited by fucose gels as well as free fucose (Figure 4A). With more than 5 mg/mL of fucose gel, a much higher gel concentration was needed for complete inhibition of LecB binding to mucin compared to melibiose gels inhibiting LecA binding to thyroglobulin. Moreover, no increase in binding signal at low gel concentration was observed despite the tetrameric structure of LecB. The IC_50_ values for the fucose gels to inhibit LecB binding to mucin are about 0.4–0.5 mg/mL and therefore markedly higher than the IC_50_ values for melibiose gels to inhibit LecA binding to thyroglobulin (Figure 3). Moreover, IC_50_ values of FG‐I and FG‐II calculated in mm based on sugar content are even 40‐fold higher than those of free fucose. The gels were synthesized from fucose monomers, where fucose is linked via a β‐glycosidic bond. As all reported ligands for LecB are α‐fucosidic or c‐fucosidic [13, 15, 49], the β‐bound fucose in the glycogels, here, could be the reason for low binding. For methyl‐ and 4‐nitrophenyl‐β‐l‐fucose, an over 750‐times and 5.5‐times, respectively, lower inhibition potency compared to the α‐counterpart was reported [50]. In another study, methyl‐β‐L‐fucoside was over 500‐fold worse than its alpha‐linked anomer [51]. So far, no multivalent β‐fucose ligands have been considered in binding assays with LecB. From this study, a multivalent effect could be concluded, although we could not compare it with monovalent β‐linked fucose. As stated above, it is known from literature that LecB binds β‐linked fucoside 500‐ or 750‐fold reduced than α‐linked fucoside and 150‐ or 400‐fold worse than free fucose [50, 51]. The multivalent β‐fucose glycogels, here, exhibit a 40‐times lower inhibition potency than free fucose, meaning an assumed 4‐ to 10‐fold improved binding compared to monovalent β‐linked fucoside from the literature. Another explanation could be the low accessibility of the sugar moiety because fucose is presented as a monosaccharide, which is located near the gel backbone. Probably, a certain distance is needed, as it is several times reported that the length of a spacer has an influence on lectin binding [52, 53, 54]. In comparison to the melibiose gels, the fucose gels are three to four times larger. The size may also affect the binding potency, as at the same concentration, the accessible surface and the amount of particles are smaller for larger gels. Control gels with melibiose and lactose showed no inhibitory effect on LecB binding, proving fucose selectivity of the lectin (Figure 4B,C).

Pure pNIPAM gel G‐I had no influence on the binding of both lectins (Figure 2C and 4C). Thus, inhibition was mediated by the sugar moiety of the gels.

### 2.3 Effect on *Pseudomonas aeruginosa* Biofilm

*P. aeruginosa* forms biofilms as a survival strategy in response to environmental and host‐related factors. It is known that lectins of *P. aeruginosa*, LecA and LecB, play an important role in biofilm formation. As in this study, synthesized glycogels inhibit the binding of both lectins, they can also affect *P. aeruginosa* biofilm. The influence on biofilm was tested in two different ways to take both into account: direct inhibition of biofilm and displacement of already formed biofilm. Interestingly, both approaches resulted in completely different effects on the biofilm.

**FIGURE 5:**
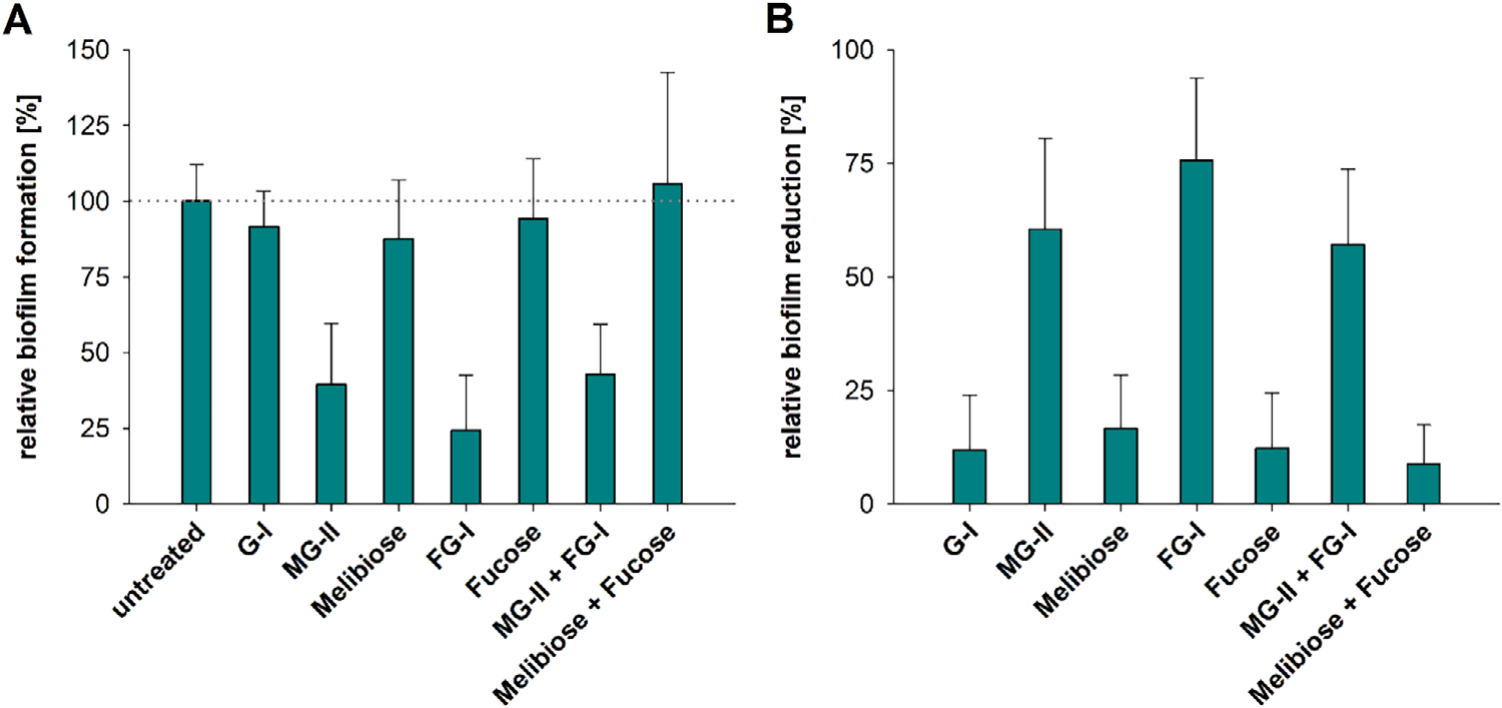
Formation (A) and reduction (B) of *P. aeruginosa* biofilm in the presence of 5 mg/mL of free saccharides, 5 mg/mL of control PNiPAm nanogel (G‐I), and 5 mg/mL of glycogels containing either fucose (FG‐I) or melibiose (MG‐II) in relation to biofilm of untreated cells. The test compounds were added before starting biofilm formation. Biofilm was stained with crystal violet. The biofilm reduction (B) is the difference between the biofilm formation value (A) and 100% and emphasizes the potency of the glycogels. The presented data are means of at least sextuplets ± standard deviation, measured in at least two independent experiments.

For the direct biofilm inhibition, the glycogels were added simultaneously with the *P. aeruginosa* culture. The strength of the overnight formed biofilm in the presence of glycogels MG‐II, FG‐I, as well as the combination thereof, control gel G‐I, melibiose, fucose as well and the combination of both sugars was compared to the untreated biofilm (Figure 5A). G‐I, as well as the soluble saccharides, did not significantly render the biofilm. In contrast, significantly lower biofilm was formed when the glycogels were present in the culture. Both glycogels inhibited the biofilm. Interestingly, the combination of both glycogels did not yield the lowest biofilm formation and performed similarly to the melibiose gel alone. When calculating the relative biofilm reduction (Figure 5B), it becomes clear that the glycogels can reduce the biofilm by up to 75%, while the control gel, as well as saccharides, reduce the biofilm by a maximum 10%–15%. These results emphasize the impact of the synthesized melibiose and fucose glycogels on *P. aeruginosa* biofilm and how important multivalent glycan presentation is. Although the inhibition potency of MG‐II to LecA is significantly higher than the inhibition potency of FG‐I to LecB (Figure 4), FG‐I seemed to affect the biofilm slightly but not significantly more than MG‐II. This may conclude that LecB may play a bigger role in *P. aeruginosa* biofilm formation than LecA. While both LecA and LecB are essential for biofilm formation, the role of LecB in matrix stabilization and interactions with exopolysaccharides like Psl may make it slightly more critical for biofilm robustness [55]. Moreover, a LecB‐deficient *P. aeruginosa* showed no biofilm‐forming ability [10]. However, the contributions of LecA to adhesion and host‐pathogen interactions are equally important in the broader context of biofilm‐associated infections [56].

Contrary results were achieved when the glycogels were added with a delay of 6 h, where *P. aeruginosa* biofilm formation was already in progress.

**FIGURE 6:**
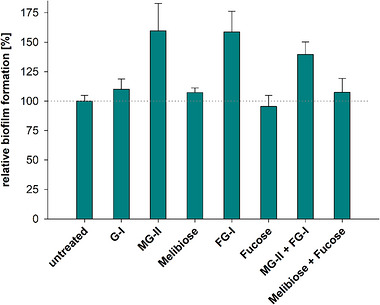
Biofilm formation of *P. aeruginosa* in the presence of 5 mg/mL of free saccharides, 5 mg/mL of control PNiPAm nanogel (G‐I), and 5 mg/mL of glycogels containing either fucose (FG‐I) or melibiose (MG‐II) in relation to biofilm of untreated cells. The test compounds were added 6 h after starting biofilm formation. Biofilm was stained with crystal violet. The presented data are means of at least sextuplets ± standard deviation, measured in at least two independent experiments.

Free saccharides as well as the PNiPAm control gel showed no significant change in biofilm formation, indicating no impact on the biofilm mode of growth. Around 100% of biofilm biomass was reached compared to untreated biofilm (Figure 6). FG‐I and MG‐II glycogels instead yielded a biofilm increase of approximately 60%, the combination of both glycogels caused with 140% a slightly less biofilm increase. It seems that the multivalent presentation of galactose and fucose mediates as a crosslinker between the already surface‐bound bacteria and bacteria in culture. Thus, further attachment of bacteria to the existing biofilm is maximized, resulting in improved biofilm formation.

In this study, identical concentrations of galactose and fucose glycogels were used to alter the biofilm formation of *P. aeruginosa* when applied to two different starting situations: 1) before biofilm was formed, and 2) after 6 h of biofilm formation. Both glycogels act as effective inhibitors when they are present before biofilm formation starts, reducing biofilm by up to 75%. In contrast, the addition of glycogels to already existing biofilm allows bacteria to aggregate much more easily by binding to these multivalent structures and enhancing the biofilm by up to 60%. These complementary results show that the time point at which the inhibitor is added has a high impact on how the biofilm of *P. aeruginosa* is altered. It is not to be assumed that glycogels influence bacterial growth. The latter glycogel addition yields more biofilm, showing no growth inhibition effect. Thus, the biofilm inhibition potency is not caused by the growth reduction of *P. aeruginosa*.

The formation and alteration of *P. aeruginosa* biofilms are significantly influenced by carbohydrate‐based compounds, which can act as both inhibitors and enhancers depending on their structure, concentration, and interaction with bacterial components, as well as the design of the biofilm assay. These dual effects highlight the complexity of targeting biofilms with carbohydrate‐derived molecules. Carbohydrate‐based compounds have shown promise in disrupting *P. aeruginosa* biofilms by targeting key structural components and bacterial adhesins. For instance, glycoclusters functionalized with galactosylated or fucosylated moieties effectively inhibit biofilm formation by interfering with the bacterial lectins LecA and LecB, which are critical for biofilm integrity. These compounds disrupt bacterial aggregation and adhesion, with fucosylated glycoclusters showing particularly strong anti‐biofilm activity [20, 21]. LecA‐binding polysaccharides, such as potato galactan and guar galactomannan, inhibit biofilm formation in a dose‐dependent manner by directly binding to LecA [57]. Similarly, sialylated glycopeptides, such as derivatives of MUC7, inhibit biofilm formation by up to 44%, underscoring the role of glycosylation in modulating bacterial interactions [58].

Alginate oligosaccharides disrupt the EPS matrix by reducing polysaccharides and extracellular DNA, which are key structural components. This leads to a significant reduction in biofilm biomass and thickness, as well as enhanced antibiotic penetration [59]. Moreover, when EPS components were enzymatically degraded, biofilm stability can be reduced by up to 69% [4]. Another effective strategy involves the use of carbohydrate amphiphiles, which mimic natural surfactants and disrupt biofilm formation by lowering surface tension and promoting bacterial motility [60]. Cationic antimicrobial polymers functionalized with glyco units (among others fucose, lactose, melibiose) demonstrated significant antimicrobial activity against *P. aeruginosa* [30]. However, while sugar incorporation into the polymers improved biocompatibility and offers potential for selective targeting, the binding to *P. aeruginosa* was, in this case, predominantly driven by electrostatic interactions rather than sugar‐lectin specificity.

Besides multivalent carbohydrate structures and oligosaccharides, monovalent high‐affinity compounds are also reported to efficiently block biofilm formation by inhibiting LecB [45, 61]. These compounds are optimized glycomimetic C‐glycosides that turn ineffective natural carbohydrate ligands into potent inhibitors without affecting bacterial viability. Presenting them in a multivalent way could enhance their effectiveness further.

Certain carbohydrate‐based compounds were demonstrated to enhance biofilm formation under specific conditions. For fucosylated and galactosylated tetravalent glycoclusters, it was observed that, while high concentrations disrupt biofilm formation and bacterial adhesion, intermediate concentrations (100–500 µm) led to increased bacterial aggregation, which could indirectly promote biofilm formation [21]. This effect was particularly pronounced with fucosylated glycoclusters, which induced aggregation even in LecB‐deficient mutants, suggesting the involvement of other bacterial adhesins. The aggregation was hypothesized to result from competing binding events with unidentified bacterial targets, highlighting the complexity of carbohydrate‐bacteria interactions. Similarly, it was reported that certain carbohydrate amphiphiles promoted biofilm formation at concentrations of 0.5 mg/mL and above [60]. This effect was attributed to the formation of rod‐like aggregates by the amphiphile with a galactose homopolymer headgroup, which may mimic fibrillar adherence proteins that facilitate biofilm development. As amphiphiles with galactose‐ and glucose‐derived random copolymer headgroups showed effective biofilm inhibition, the galactose density and distance of presented galactose units may play a role in bacterial aggregation.

The glycogels used in this study are highly decorated with terminal galactose and fucose residues, ensuring high binding affinities to LecA and LecB. These structures block both bacterial lectins and reduce their ability to build up biofilm. However, when biofilm formation has already started, glycogels act as a multivalent crosslinker that multiplies the binding sites on the already existing biofilm. This leads to increased bacterial attachment and stronger biofilm formation.

The dual role of carbohydrate‐based compounds in inhibiting and enhancing biofilm formation underscores the need for precise dosing and a thorough understanding of their mechanisms of action. While these compounds hold potential as anti‐biofilm agents, their unintended effects on biofilm enhancement highlight the complexity of biofilm dynamics and the challenges in developing effective therapies. Future research should focus on optimizing the structure and concentration of carbohydrate‐based compounds to maximize their inhibitory effects while minimizing the risk of biofilm promotion.

## 3 Conclusion

The synthesized glycosylated nanogels, presenting melibiose and fucose, exhibit distinct inhibitory effects on the binding of *P. aeruginosa* lectins, LecA and LecB, which are critical for biofilm formation. Competitive inhibition assays revealed that melibiose gels are potent inhibitors of LecA binding to thyroglobulin, while fucose gels effectively inhibit LecB binding to mucin. In direct comparison, the fucose nanogels demonstrate a markedly lower efficacy compared to the melibiose gels. The results, however, highlight the effect in lectin interactions that even non‐optimal ligands can achieve good inhibition results if presented multivalently.

However, both types of glycogels demonstrate significant potential in influencing biofilm formation. Pre‐treatment with these glycogels effectively inhibited biofilm development, achieving up to 75% reduction, while their application after biofilm initiation led to a surprising increase in biofilm biomass by approximately 60%, highlighting their role as multivalent crosslinkers. These findings underscore the complexity of targeting biofilms with carbohydrate‐based compounds, where their structural design and application timing critically determine their inhibitory or enhancing effects, emphasizing the need for careful consideration in therapeutic strategies aimed at managing biofilm‐associated infections. This research contributes valuable insights into the role of lectin‐targeting compounds in biofilm management, paving the way for innovative therapeutic approaches to combat infections caused by biofilm‐forming bacteria.

## 4 Experimental Section

### 4.1 Materials

All chemicals were purchased from commercial sources. We recrystallized *N‐*isopropylacrylamide (NiPAm; 97%, Sigma–Aldrich) from n‐hexane. Water was double‐deionized by a Milli‐Q purification system (18.2 MΩ cm, Biopak Polisher). Dichloromethane (CH_2_Cl_2_; for synthesis) and chloroform (CHCl_3_; extra pure) were redistilled before use. *N*,*N*’‐Methylenebis(acrylamide) (MBA; 99%, Sigma–Aldrich), 4,4′‐azobis(4‐cyanovaleric acid) (ACVA; ≥98%, Sigma–Aldrich), D(+)‐melibiose monohydrate (Mel; ≥99%, Roth), L‐(‐)‐fucose (Fuc; ≥99%, Sigma–Aldrich), D‐lactose monohydrate (Lac; Carbosynth), ammonium carbonate ((NH_4_)_2_CO_3_; ≥30.5% NH_3_, extra pure, Roth), dimethyl sulfoxide (DMSO; VWR), methacryloyl chloride (purum, dist., ≥97%, Sigma–Aldrich), sodium carbonate (≥99%, anhydrous, Roth), tetrahydrofuran (THF; p. a., Chemsolute), acetonitrile (≥99,8%, for preparative HPLC, Roth), hydroquinone (99.5%, Acros Organics), diethylether (Et_2_O; p. a., Chemsolute), methanol (MeOH; extra pure), silica gel (high‐purity grade, pore size 60 Å, Sigma–Aldrich), sodium dodecyl sulfate (SDS; ≥99,5%, blotting‐grade, Roth) were used as received.

### 4.2 Synthesis of Glycomonomers

The glycosyl methacrylamides were synthesized according to a previously published work [46, 62]. The two‐step synthesis involves the amination of saccharides followed by methacrylation of the glycosylamines and leads to glycomonomers in their β‐configuration.

Briefly, the respective saccharide was dissolved in a solvent, and ammonium carbonate was added (Table 2). The reaction mixture was heated in the microwave reactor (START 1500 rotaPREP, MSL, Leutkirch, Germany) for 90 min under stirring. After the mixture had cooled down, the ammonium carbonate and the solvent were removed by rotary evaporation at 40°C under reduced pressure. In the case of LacNH_2_, the glycosylamine was precipitated in 40 mL of MeOH after reaction and dried. The crude products were dried in high vacuum and stored at 4°C.

**TABLE 2:** Synthesis details of glycosylamines.

	m(Saccharide) [g]	n(Saccharide) [mmol]	Solvent	V(Solvent) [mL]	m(Ammonium carbonate) [g]	T [°C]
MelNH_2_	5.0	13.9	H_2_O	100	50	40
FucNH_2_	2.0	12.0	MeOH	197	9.85	60
LacNH_2_	3.3	8.33	DMSO	12.0	5.0	40

The respective glycosylamine was dissolved in a mixture of methanol and Milli‐Q water (1:1) (Table 3). Sodium carbonate was added, and the reaction mixture was cooled in an ice‐water bath. Methacryloyl chloride in THF was added dropwise within 10–30 min. The reaction mixture was stirred for a further 10–60 min in the ice‐water bath or at room temperature before the volatile solvents were removed by rotary evaporation at 30°C. The crude product was purified by silica gel column chromatography (MelMAm: acetonitrile/H_2_O 9:1 → 4:1; FucMAm: CH_2_Cl_2_/MeOH 5:1; LacMAm: acetonitrile/H_2_O 9:1). LacMAm was additionally washed with Et_2_O. MelMAm was stabilized with hydroquinone (3 ppm) to prevent spontaneous polymerization. The compounds were analyzed by ^1^H‐NMR and ESI‐MS and compared with published data.

**TABLE 3:** Synthesis details of glycomonomers.

	n(Glcosylamine) [mmol]	V(Solvent mixture) [mL]	n(Sodium carbonate) [mmol]	n(Methacryloyl chloride) [mmol]	V(THF) s[mL]
MelMAm	13.9	150	77.8	42.6	35
FucMAm	11.4	124	63.7	39.7	33
LacMAm	3.17	16.4	12.7	9.50	6.3

LacMAm. ^1^H‐NMR (D_2_O, 300 MHz): δ 5.83 (s, 1 H), 5.53–5.67 (m, 1 H), 5.12 (d, ^3^J = 9.2 Hz, 1 H), 4.51 (d, ^3^J = 7.7 Hz, 1 H), 3.53–4.01 (m, 12 H), 1.99 (s, 3 H); ESI MS, m/z calcd for C_16_H_28_NO_11_: [M + Na]^+^ 432.38, found: 432.11 [M + Na]^+^. MelMAm. ^1^H‐NMR (300 MHz, D_2_O): δ = 5.81 (s, 1 H), 5.59 (d, ^3^J = 1.6 Hz, 1 H), 5.08 (d, ^3^J = 8.9 Hz, 1 H), 5.00 (d, ^3^J = 3.5 Hz, 1 H), 3.45–4.05 (m, 12 H), 1.98 (s, 3 H); ESI MS, calcd for C_16_H_27_NO_11_: [M + Na]^+^ 432.38, found: 432.10 [M + Na]^+^. FucMAm. 1H‐NMR (300 MHz, D_2_O): δ = 5.69 (1 H, s), 5.47 (1 H, s), 4.90 (1 H, d, 3J = 8.5 Hz), 3.82 (1 H, q, 3J = 6.5 Hz), 3.59–3.80 (3 H, m), 1.86 (3 H, s), 1.15 (3 H, d, 3J = 6.5 Hz); ESI MS, calcd for C_10_H_17_NO_5_: [M + Na]^+^ 254.24, found: 254.04 [M + Na]^+^.

Spectra are presented in the supporting information.

### 4.3 Synthesis of Nanogels

Nanogels were synthesized according to a previously published procedure [46]. Glycogels were abbreviated with the first letter of the respective saccharide to MG, FG, and LG. Briefly, the monomers, crosslinker, SDS, and initiator were dissolved in Milli‐Q water and purged with nitrogen for 30 min (Table 4). The reaction was started by submerging the reaction flask in an 80°C oil bath. In the case of MG‐II, an additional initiator (Χ_2_(ACVA)) was added after 2 h. After we let the reaction proceed, the reaction mixture was allowed to cool down. Nanogels were dialyzed against deionized water and lyophilized afterward to obtain a white solid. Large aggregates were removed by filtering through Kimtech Science precision wipes (Darmstadt, Germany) before freeze‐drying. Pure PNiPAm nanogels were synthesized similarly, but the reaction mixture was purged at 80°C without the initiator. After equilibration, the reaction was started by the addition of the initiator.

**TABLE 4:** Synthesis details of nanogels.^a)^

Nanogel	n(Glyco‐monomer) [mmol]	n(NiPAm) [mmol]	V(H_2_O) [mL]	Χ(MBA) [mol%]	c(SDS) [mmol/L]	Χ_1_(ACVA) [mol%]	Χ_2_(ACVA) [mol%]	t [h]
G‐I	—	5.0	50	5.0	0.2	0.25	—	4.0
MG‐I	0.1	0.4	5.0	10	0.4	3.00	—	22
MG‐II	0.1	0.4	5.0	10	0.4	1.00	2.00	17
FG‐I	0.3	1.2	15	10	0.4	1.00	—	4.0
FG‐II	0.3	1.2	15	5.0	0.4	1.00	—	4.0
LG‐I	0.1	0.4	5.0	5.0	0.2	2.00	—	20

^a)^ The molfraction Χ refers to the total monomer amount.

### 4.4 Dynamic Light Scattering

The hydrodynamic diameters and PDIs were investigated by using dynamic light scattering (DLS) (Malvern Zetasier Nano‐ZS, Kassel, Germany). Measurements were performed in disposable polymethylmethacrylate cuvettes at a backscattering angle of 173° five times and at 20°C or 50°C. For the measurements at 20°C, we let the samples equilibrate for 5 min, and for the measurements at 50°C, the samples were allowed to equilibrate for 10 min to ensure complete collapse of the glycogels.

### 4.5 Phenol‐Sulfuric Acid Assay for Determination of Total Sugar Content

The phenol‐sulfuric acid assay was performed as described elsewhere [46]. Briefly, 50 µL of fucose nanogels with concentrations in the range of 2–5 mg/mL were mixed with 150 µL sulfuric acid (95%, Th. Geyer, Renningen, Germany), and 30 µL of 5% phenol (Sigma–Aldrich) was subsequently added, followed by mixing. The mixture was incubated at 90°C for 5 min and allowed to cool down in a water bath for a further 5 min. After transferring the solution into a 96‐well plate (Carl Roth), the absorption at 490 nm was measured. Fucose was used separately for calibration and control gels without sugar to prove the suitability of the assay.

### 4.6 Lectin Studies

Binding studies were done with biotin‐labeled *P. aeruginosa* lectins LecA and LecB (both from Elicityl). The lectin binding to the nanogels was proven by an ELISA‐type competitive inhibition assay, similar to previously described assays [46, 63, 64]. Glycogels were used as inhibitors of lectin binding to immobilized glycoproteins. In a previous binding assay with different immobilized glycoproteins and varying lectin concentrations, it was found that LecA shows good binding to thyroglobulin and LecB to mucin from porcine stomach.

In microtiter plates (MaxiSorp, Nunc, Wiesbaden, Germany), thyroglobulin (100 µL of 5 µg/mL bovine thyroglobulin (Sigma–Aldrich)) or mucin (100 µL of 100 µg/mL porcine stomach mucin (Sigma–Aldrich), both in sodium carbonate buffer pH 9.6) was immobilized overnight. After washing with PBS‐Tween (0.05% (v/v) Tween‐20), residual binding sites were blocked with 2% BSA (bovine serum albumin, Carl Roth) in PBS supplemented with 0.1 mm CaCl_2_. Wells were washed three times with PBS‐Ca‐Tween. Varying concentrations of inhibitor and 10 µg/mL of lectin were incubated simultaneously for 1 h. Controls without inhibitor and without lectin were performed to indicate minimal and maximal binding, respectively. Wells were again washed with PBS‐Ca‐Tween buffer and incubated with Atto488‐labelled streptavidin (Sigma–Aldrich, 1:1000 in PBS‐Ca) for 1 h. The fluorescence was read out at 488/520 nm and indicated indirectly the amount of residual lectin. Measured data were analyzed using Sigma Plot (Systat software GmbH, 11.0, Erkrath, Germany).

### 4.7 Biofilm Assay with *P. aeruginosa*

*Pseudomonas aeruginosa* DSM 50071 strain was cultivated in lysogeny broth at 37°C and 120 rpm until the stationary phase was reached, followed by the dilution of culture in M9 medium to an OD_600_ of 0.06 or 0.1, respectively. M9 medium contains 48 mm Na_2_HPO_4_, 22 mm KH_2_PO_4_, 9 mm NaCl, 19 mm NH_4_Cl, 2 mm MgSO_4_, 100 µm CaCl_2_, pH 7.2 with 0.4% glucose as the sole carbon source. The biofilm formation was performed in a sterile 96‐well plate with a flat bottom (VWR or Eppendorf) in two different set‐ups.

Set‐up 1: In a 96‐well plate, 10 µL of the sample dissolved in M9 medium (50 mg/mL) was mixed with 90 µL bacteria suspension (OD_600_ = 0.06) and incubated at 37°C and 5% CO_2_ overnight.

Set‐up 2: 100 µL bacteria suspension (OD_600_ = 0.1) was incubated for 6 h at 37°C. Afterward, 20 µL were removed from each well and replaced with 20 µL sample. The samples were dissolved in M9 medium to a concentration of 25 mg/mL and homogenized by ultrasonic sound at 22°C, 100% power for 30 min. The wells were further cultivated overnight at 30°C.

For analysis of both setups, the bacterial culture was removed, and wells were washed twice with 200 µL Milli‐Q water. Afterward, dried wells were incubated with 150 µL of 0.1% crystal violet dye for 20 min at ambient temperature. Wells were washed again twice with 200 µL Milli‐Q water to remove non‐bound dye. The dye that was bound to biofilm was dissolved in 150 µL of 33% acetic acid, and 100 µL was measured at 590 nm for quantitative analysis. Data from samples incubated in medium were subtracted from samples incubated in culture. The presented data are means of at least sextuplets ± standard deviation, measured in at least two independent experiments and in relation to the negative control (biofilm formation when adding water instead of glycogels).

## Conflicts of Interest

The authors declare no conflicts of interest.

## Supporting information




**Supporting File**: marc70158‐sup‐0001‐SuppMat.docx.

## Data Availability

The data that support the findings of this study are available from the corresponding author upon reasonable request.

## References

[1] J. A. Driscoll , S. L. Brody , and M. H. Kollef , “The Epidemiology, Pathogenesis and Treatment of Pseudomonas aeruginosa Infections,” Drugs 67, no. 3 (2007): 351–368, 10.2165/00003495-200767030-00003. 17335295

[2] E. Tacconelli , E. Carrara , A. Savoldi , et al., “Discovery, Research, and Development of New Antibiotics: The WHO Priority List of Antibiotic‐resistant Bacteria and Tuberculosis,” The Lancet Infectious Diseases 18, no. 3 (2018): 318–327, 10.1016/S1473-3099(17)30753-3. 29276051

[3] M. T. T. Thi , D. Wibowo , and B. H. A. Rehm , “Pseudomonas aeruginosa Biofilms,” International Journal of Molecular Sciences 21, no. 22 (2020): 8671, 10.3390/ijms21228671. 33212950 PMC7698413

[4] A. Vetrivel , M. Ramasamy , P. Vetrivel , et al., “Pseudomonas aeruginosa Biofilm Formation and Its Control,” Biologics 1, no. 3 (2021): 312–336, 10.3390/biologics1030019.

[5] C. Gozali and C. R. Tjampakasari , “Pseudomonas aeruginosa Biofilm Formation and Its Resistance to Beta‐lactam Antibiotics,” Damianus Journal of Medicine 22, no. 2 (2023): 162–172, 10.25170/djm.v22i2.3928.

[6] C.‐Y. Chang , “Surface Sensing for Biofilm Formation in Pseudomonas aeruginosa,” Frontiers in Microbiology 8 (2017): 2671, 10.3389/fmicb.2017.02671. 29375533 PMC5767216

[7] K. Winzer , C. Falconer , N. C. Garber , S. P. Diggle , M. Camara , and P. Williams , “The Pseudomonas aeruginosa Lectins PA‐IL and PA‐IIL Are Controlled by Quorum Sensing and by RpoS,” Journal of Bacteriology 182, no. 22 (2000): 6401–6411, 10.1128/JB.182.22.6401-6411.2000. 11053384 PMC94786

[8] M. E. Mattmann and H. E. Blackwell , “Small Molecules That Modulate Quorum Sensing and Control Virulence in Pseudomonas aeruginosa,” The Journal of Organic Chemistry 75, no. 20 (2010): 6737–6746, 10.1021/jo101237e. 20672805 PMC2952040

[9] C. Chemani , A. Imberty , S. de Bentzmann , et al., “Role of LecA and LecB Lectins in Pseudomonas aeruginosa‐induced Lung Injury and Effect of Carbohydrate Ligands,” Infection and Immunity 77, no. 5 (2009): 2065–2075, 10.1128/IAI.01204-08. 19237519 PMC2681743

[10] D. Tielker , S. Hacker , R. Loris , et al., “Pseudomonas aeruginosa Lectin LecB Is Located in the Outer Membrane and Is Involved in Biofilm Formation,” Microbiology (Reading, England) 151 (2005): 1313–1323, 10.1099/mic.0.27701-0. 15870442

[11] A. Imberty , M. Wimmerová , E. P. Mitchell , and N. Gilboa‐Garber , “Structures of the lectins From Pseudomonas aeruginosa: Insights Into the molecular basis for host glycan recognition,” Microbes and Infection 6, no. 2 (2004): 221–228, 10.1016/j.micinf.2003.10.016. 15049333

[12] S. Behren and U. Westerlind , “Novel Approaches To Design Glycan‐Based Antibacterial Inhibitors,” European Journal of Organic Chemistry 26, no. 1 (2023): 26, 10.1002/ejoc.202200795.

[13] J.‐L. Reymond , M. Bergmann , and T. Darbre , “Glycopeptide Dendrimers as Pseudomonas aeruginosa Biofilm Inhibitors,” Chemical Society Reviews 42, no. 11 (2013): 4814–4822, 10.1039/c3cs35504g. 23370573

[14] S. Wagner , R. Sommer , S. Hinsberger , et al., “Novel Strategies for the Treatment of Pseudomonas aeruginosa Infections,” Journal of Medicinal Chemistry 59, no. 13 (2016): 5929–5969, 10.1021/acs.jmedchem.5b01698. 26804741

[15] M. B. Calvert , V. R. Jumde , and A. Titz , “Pathoblockers or Antivirulence Drugs as a New Option for the Treatment of Bacterial Infections,” Beilstein Journal of Organic Chemistry 14 (2018): 2607–2617, 10.3762/bjoc.14.239. 30410623 PMC6204809

[16] M. S. Majik and P. T. Parvatkar , “Next Generation Biofilm Inhibitors for Pseudomonas aeruginosa: Synthesis and Rational Design Approaches,” Current Topics in Medicinal Chemistry 14, no. 1 (2014): 81–109, 10.2174/1568026613666131113152257. 24236724

[17] A. A. M. Kamal , C. K. Maurer , G. Allegretta , J. Haupenthal , M. Empting , and R. W. Hartmann , “Quorum Sensing Inhibitors as Pathoblockers for Pseudomonas aeruginosa Infections: A New Concept in Anti‐Infective Drug Discovery,” Antibacterials, eds. J. F. Fisher , S. Mobashery , M. J Miller (Springer International Publishing, 2018): pp. 185–210.

[18] R. U. Kadam , M. Bergmann , M. Hurley , et al., “A Glycopeptide Dendrimer Inhibitor of the Galactose‐Specific Lectin LecA and of Pseudomonas aeruginosa Biofilms,” Angewandte Chemie International Edition 50, no. 45 (2011): 10631–10635, 10.1002/anie.201104342. 21919164 PMC3262149

[19] M. Bergmann , G. Michaud , R. Visini , et al., “Multivalency Effects on Pseudomonas aeruginosa Biofilm Inhibition and Dispersal by Glycopeptide Dendrimers Targeting Lectin LecA,” Organic & Biomolecular Chemistry 14, no. 1 (2016): 138–148, 10.1039/c5ob01682g. 26416170

[20] E. M. V. Johansson , S. A. Crusz , E. Kolomiets , et al., “Inhibition and Dispersion of Pseudomonas aeruginosa Biofilms by Glycopeptide Dendrimers Targeting the Fucose‐specific Lectin LecB,” Chemistry & Biology 15, no. 12 (2008): 1249–1257, 10.1016/j.chembiol.2008.10.009. 19101469

[21] A. M. Boukerb , A. Rousset , N. Galanos , et al., “Antiadhesive Properties of Glycoclusters Against Pseudomonas aeruginosa Lung Infection,” Journal of Medicinal Chemistry 57, no. 24 (2014): 10275–10289, 10.1021/jm500038p. 25419855

[22] G. M. Consoli , G. Granata , V. Cafiso , S. Stefani , and C. Geraci , “Multivalent Calixarene‐based C‐fucosyl Derivative: A New Pseudomonas aeruginosa Biofilm Inhibitor,” Tetrahedron Letters 52, no. 44 (2011): 5831–5834, 10.1016/j.tetlet.2011.08.142.

[23] M. Taouai , K. Chakroun , R. Sommer , et al., “Glycocluster Tetrahydroxamic Acids Exhibiting Unprecedented Inhibition of Pseudomonas aeruginosa Biofilms,” Journal of Medicinal Chemistry 62, no. 17 (2019): 7722–7738, 10.1021/acs.jmedchem.9b00481. 31449405

[24] S. Wang , L. Dupin , M. Noël , et al., “Toward the Rational Design of Galactosylated Glycoclusters That Target Pseudomonas aeruginosa Lectin A (LecA): Influence of Linker Arms That Lead to Low‐Nanomolar Multivalent Ligands,” Chemistry—A European Journal 22, no. 33 (2016): 11785–11794, 10.1002/chem.201602047. 27412649

[25] C. Ligeour , L. Dupin , A. Marra , et al., “Synthesis of Galactoclusters by Metal‐Free Thiol “Click Chemistry” and Their Binding Affinities for Pseudomonas aeruginosa Lectin LecA,” European Journal of Organic Chemistry 2014, no. 34 (2014): 7621–7630, 10.1002/ejoc.201402902.

[26] B. Gerland , A. Goudot , G. Pourceau , et al., “Synthesis of a Library of Fucosylated Glycoclusters and Determination of Their Binding Toward Pseudomonas aeruginosa Lectin B (PA‐IIL) Using a DNA‐based Carbohydrate Microarray,” Bioconjugate Chemistry 23, no. 8 (2012): 1534–1547, 10.1021/bc2006434. 22799498

[27] M. Smadhi , S. D. Bentzmann , A. Imberty , M. Gingras , R. Abderrahim , and P. G. Goekjian , “Expeditive Synthesis of Trithiotriazine‐cored Glycoclusters and Inhibition of Pseudomonas aeruginosa Biofilm Formation,” Beilstein Journal of Organic Chemistry 10 (2014): 1981–1990, 10.3762/bjoc.10.206. 25246957 PMC4168900

[28] C. O'Reilly , S. Blasco , B. Parekh , et al., “Ruthenium‐centred Btp Glycoclusters as Inhibitors for Pseudomonas aeruginosa Biofilm Formation,” RSC Advances 11, no. 27 (2021): 16318–16325, 10.1039/D0RA05107A. 35479152 PMC9030604

[29] L. Malinovská , S. T. Le , M. Herczeg , et al., “Synthesis of β‐d‐galactopyranoside‐Presenting Glycoclusters, Investigation of Their Interactions with Pseudomonas aeruginosa Lectin A (PA‐IL) and Evaluation of Their Anti‐Adhesion Potential,” Biomolecules 9 (2019): 686, 10.3390/biom9110686. 31683947 PMC6920806

[30] A. M. Bapolisi , S. Tank , A.‐C. Lehnen , et al., “Sugars in Antimicrobial Polymers—The Impact of Sugar‐Lectin Binding in Membrane Interaction of Cationic Glycopolymers,” Adv Materials Inter 12, no. 16 (2025): 00273, 10.1002/admi.202500273.

[31] S. Kirkeby and D. Moe , “Analyses of Pseudomonas aeruginosa Lectin Binding to α‐Galactosylated Glycans,” Current Microbiology 50, no. 6 (2005): 309–313, 10.1007/s00284-005-4484-y. 15968505

[32] S. Kirkeby , A. K. Hansen , A. d'Apice , and D. Moe , “The Galactophilic Lectin (PA‐IL, gene LecA) From Pseudomonas aeruginosa. Its Binding Requirements and the Localization of Lectin Receptors in Various Mouse Tissues,” Microbial Pathogenesis 40, no. 5 (2006): 191–197, 10.1016/j.micpath.2006.01.006. 16542817

[33] J. J. Lundquist and E. J. Toone , “The Cluster Glycoside Effect,” Chemical Reviews 102, no. 2 (2002): 555–578, 10.1021/cr000418f. 11841254

[34] A. Angeli , M. Li , L. Dupin , et al., “Design and Synthesis of Galactosylated Bifurcated Ligands With Nanomolar Affinity for Lectin LecA From Pseudomonas aeruginosa,” Chembiochem 18, no. 11 (2017): 1036–1047, 10.1002/cbic.201700154. 28318079

[35] S. Wagner , D. Hauck , M. Hoffmann , et al., “Covalent Lectin Inhibition and Application in Bacterial Biofilm Imaging,” Angewandte Chemie International Edition 56, no. 52 (2017): 16559–16564, 10.1002/anie.201709368. 28960731 PMC5767747

[36] S. Perret , C. Sabin , C. Dumon , et al., “Structural Basis for the Interaction Between human Milk Oligosaccharides and the Bacterial Lectin PA‐IIL of Pseudomonas aeruginosa,” Biochemical Journal 389, no. Pt 2 (2005): 325–332, 10.1042/BJ20050079. 15790314 PMC1175109

[37] S. Weichert , S. Jennewein , E. Hüfner , et al., “Bioengineered 2′‐Fucosyllactose and 3‐Fucosyllactose Inhibit the Adhesion of Pseudomonas Aeruginosa and Enteric Pathogens to Human Intestinal and Respiratory Cell Lines,” Nutrition Research 33, no. 10 (2013): 831–838, 10.1016/j.nutres.2013.07.009. 24074741

[38] L. C. Breitenbach Barroso Coelho , P. Marcelino dos Santos Silva , W. Felix de Oliveira , et al., “Lectins as Antimicrobial Agents,” Journal of Applied Microbiology 125, no. 5 (2018): 1238–1252, 10.1111/jam.14055. 30053345

[39] S. Bala Subramaniyan , R. Senthilnathan , J. Arunachalam , and V. Anbazhagan , “Revealing the Significance of the Glycan Binding Property of Butea Monosperma Seed Lectin for Enhancing the Antibiofilm Activity of Silver Nanoparticles Against Uropathogenic Escherichia coli,” Bioconjugate Chemistry 31, no. 1 (2020): 139–148, 10.1021/acs.bioconjchem.9b00821. 31860279

[40] S. Nsayef Muslim , A. N. Mohammed Ali , and I. G. Auda , “Anti‐biofilm and anti‐virulence effects of silica oxide nanoparticle–conjugation of lectin purified From Pseudomonas aeruginosa,” IET Nanobiotechnology 15, no. 3 (2021): 318–328, 10.1049/nbt2.12022. 34694672 PMC8675845

[41] N. Bodenberger , D. Kubiczek , D. Halbgebauer , et al., “Lectin‐Functionalized Composite Hydrogels for "Capture‐and‐Killing" of Carbapenem‐Resistant Pseudomonas aeruginosa",” Biomacromolecules 19, no. 7 (2018): 2472–2482, 10.1021/acs.biomac.8b00089. 29665678

[42] J. E. Kong , S. Kahkeshani , I. Pushkarsky , and D. Di Carlo , “Research Highlights: Micro‐engineered Therapies,” Lab on A Chip 14, no. 24 (2014): 4585–4589, 10.1039/c4lc90107j. 25353397

[43] A. Jans , R. R. Rosencrantz , A. D. Mandic , et al., “Glycan‐Functionalized Microgels for Scavenging and Specific Binding of Lectins,” Biomacromolecules 18, no. 5 (2017): 1460–1465, 10.1021/acs.biomac.6b01754. 28257575

[44] T. Mohy El Dine , R. Jimmidi , A. Diaconu , et al., “Pillar[5]arene‐Based Polycationic Glyco[2]rotaxanes Designed as Pseudomonas aeruginosa Antibiofilm Agents,” Journal of Medicinal Chemistry 64, no. 19 (2021): 14728–14744, 10.1021/acs.jmedchem.1c01241. 34542288

[45] R. Sommer , S. Wagner , K. Rox , et al., “Glycomimetic, Orally Bioavailable LecB Inhibitors Block Biofilm Formation of Pseudomonas aeruginosa,” Journal of the American Chemical Society 140, no. 7 (2018): 2537–2545, 10.1021/jacs.7b11133. 29272578

[46] J. O. S. J. Tang , S. Rosencrantz , L. Tepper , et al., “Functional Glyco‐Nanogels for Multivalent Interaction With Lectins,” Molecules (Basel, Switzerland) 24, no. 10 (2019): 1865, 10.3390/molecules24101865. 31096570 PMC6572176

[47] J. Rodrigue , G. Ganne , B. Blanchard , et al., “Aromatic Thioglycoside Inhibitors Against the Virulence Factor LecA From Pseudomonas aeruginosa,” Organic & Biomolecular Chemistry 11, no. 40 (2013): 6906–6918, 10.1039/C3OB41422A. 24057051

[48] G. Cioci , E. P. Mitchell , C. Gautier , et al., “Structural basis of calcium and galactose recognition by the lectin PA‐IL of Pseudomonas aeruginosa,” FEBS Letters 555, no. 2 (2003): 297–301, 10.1016/s0014-5793(03)01249-3. 14644431

[49] A. Audfray , A. Varrot , and A. Imberty , “Bacteria Love Our Sugars: Interaction Between Soluble Lectins and human Fucosylated Glycans, Structures, Thermodynamics and Design of Competing Glycocompounds,” Comptes Rendus Chimie 16, no. 5 (2013): 482–490, 10.1016/j.crci.2012.11.021.

[50] A. M. Wu , Y.‐P. Gong , C.‐C. Li , and N. Gilboa‐Garber , “Duality of the carbohydrate‐recognition system of Pseudomonas aeruginosa ‐II lectin (PA‐IIL),” FEBS Letters 584, no. 11 (2010): 2371–2375, 10.1016/j.febslet.2010.04.026. 20398656

[51] R. Sommer , S. Wagner , A. Varrot , et al., “The Virulence Factor LecB Varies in Clinical Isolates: Consequences for Ligand Binding and Drug Discovery,” Chemical Science 7, no. 8 (2016): 4990–5001, 10.1039/c6sc00696e. 30155149 PMC6018602

[52] K. Buffet , I. Nierengarten , N. Galanos , et al., “Pillar[5]arene‐Based Glycoclusters: Synthesis and Multivalent Binding to Pathogenic Bacterial Lectins,” Chemistry—A European Journal (2016), 22 (9), 2955–2963, 10.1002/chem.201504921. 26845383

[53] E. Zahorska , F. Rosato , K. Stober , et al., “Neutralizing the Impact of the Virulence Factor LecA From Pseudomonas aeruginosa on Human Cells With New Glycomimetic Inhibitors,” Angewandte Chemie International Edition 62, no. 7 (2023): 202215535, 10.1002/anie.202215535. PMC10107299 36398566

[54] K. Wojtczak and J. P. Byrne , “Structural Considerations for Building Synthetic Glycoconjugates as Inhibitors for Pseudomonas aeruginosa Lectins,” Chemmedchem 17 (2022): 202200081, 10.1002/cmdc.202200081. PMC9321714 35426976

[55] D. Passos da Silva , M. L. Matwichuk , D. O. Townsend , et al., “The Pseudomonas aeruginosa Lectin LecB Binds to the Exopolysaccharide Psl and Stabilizes the Biofilm Matrix,” Nature Communications 10, no. 1 (2019): 2183, 10.1038/s41467-019-10201-4. PMC6522473 31097723

[56] S. P. Diggle , R. E. Stacey , C. Dodd , M. Cámara , P. Williams , and K. Winzer , “The Galactophilic Lectin, LecA, Contributes to Biofilm Development in Pseudomonas aeruginosa,” Environmental Microbiology 8, no. 6 (2006): 1095–1104, 10.1111/j.1462-2920.2006.001001.x. 16689730

[57] A. Grishin , A. S. Karyagina , I. G. Tiganova , et al., “Inhibition of Pseudomonas aeruginosa Biofilm Formation by LecA‐binding Polysaccharides,” International Journal of Antimicrobial Agents 42, no. 5 (2013): 471–472, 10.1016/j.ijantimicag.2013.07.003. 23988717

[58] W. Ma , J. Luo , H. Liu , et al., “Chemoenzymatic Synthesis of Highly O ‐Glycosylated MUC7 Glycopeptides for Probing Inhibitory Activity Against Pseudomonas aeruginosa Biofilm Formation,” Angewandte Chemie International Edition 64, no. 20 (2025): 202424312, 10.1002/anie.202424312. 39996424

[59] L. C. Powell , M. F. Pritchard , E. L. Ferguson , et al., “Targeted Disruption of the Extracellular Polymeric Network of Pseudomonas aeruginosa Biofilms by Alginate Oligosaccharides,” Npj Biofilms and Microbiomes 4, no. 1 (2018): 13, 10.1038/s41522-018-0056-3. 29977590 PMC6026129

[60] E. L. Dane , A. E. Ballok , G. A. O'Toole , and M. W. Grinstaff , “Synthesis of Bioinspired Carbohydrate Amphiphiles That Promote and Inhibit Biofilms,” Chemical Science 5, no. 2 (2014): 551–557, 10.1039/C3SC52777H. PMC3873002 24376911

[61] R. Sommer , K. Rox , S. Wagner , et al., “Anti‐biofilm Agents Against Pseudomonas aeruginosa: A Structure–Activity Relationship Study of C ‐Glycosidic LecB Inhibitors,” Journal of Medicinal Chemistry 62, no. 20 (2019): 9201–9216, 10.1021/acs.jmedchem.9b01120. 31553873 PMC6873108

[62] J. S. J. Tang , K. Schade , L. Tepper , S. Chea , G. Ziegler , and R. R. Rosencrantz , “Optimization of the Microwave Assisted Glycosylamines Synthesis Based on a Statistical Design of Experiments Approach,” Molecules (Basel, Switzerland) 25, no. 21 (2020): 5121, 10.3390/molecules25215121. 33158070 PMC7663175

[63] S. Böcker , D. Laaf , and L. Elling , “Galectin Binding to Neo‐Glycoproteins: LacDiNAc Conjugated BSA as Ligand for Human Galectin‐3,” Biomolecules 5 (2015): 1671–1696, 10.3390/biom5031671. 26213980 PMC4598770

[64] S. Böcker and L. Elling , “Biotinylated N‐Acetyllactosamine‐ and,N‐Diacetyllactosamine‐Biotinylated N‐Acetyllactosamine‐ and N,N‐Diacetyllactosamine‐Based Oligosaccharides as Novel Ligands for Human Galectin‐3,” Bioengineering 4 (2017): 31, 10.3390/bioengineering4020031. 28952509 PMC5590477

