# Supplementary material for: Glyco‐Nanogels for Modulating Pseudomonas aeruginosa Biofilm
Source: Macromol Rapid Commun. 2025 Nov 29;47(14):e00807. doi: 10.1002/marc.202500807 (PMC13384796; doi:10.1002/marc.202500807)
Supplement: Supplementary file 1 — Supporting File: marc70158‐sup‐0001‐SuppMat.docx. [file MARC-47-e00807-s001.docx]

Supporting Information

Glyco-Nanogels for Modulating Pseudomonas aeruginosa Biofilm

Sophia Rosencrantz^1,2^, Jo Sing Julia Tang^1^, Karina Koenig^1,2^, Sany Chea^1^ and Ruben R. Rosencrantz^1,3^*

^1^Fraunhofer Institute for Applied Polymer Research IAP, Life Science and Bioprocesses, Geiselbergstr. 69, 14476 Potsdam, Germany

^2^Fraunhofer Cluster of Excellence Immune-Mediated Diseases CIMD, Frankfurt am Main, Germany

^3^Brandenburg University of Technology (BTU) Cottbus-Senftenberg, Institute of Materials Chemistry, Chair for Biofunctional Polymermaterials, Universitätsplatz 1, 01968 Senftenberg, Germany

* Corresponding author: ruben.rosencrantz@iap.fraunhofer.de; Tel.: +49-331-568-3203


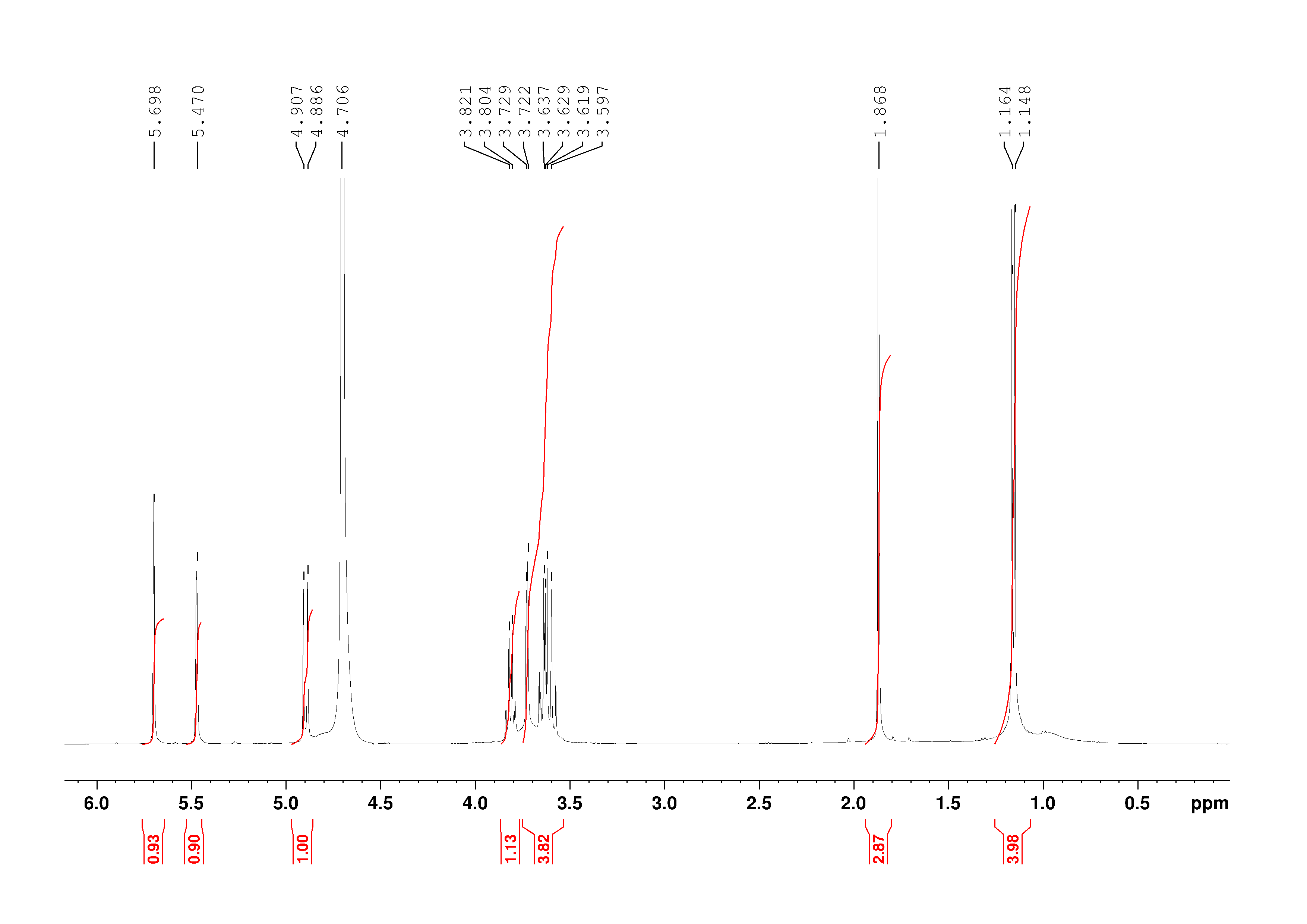


Figure S1: 1H-NMR spectra of FucMAm


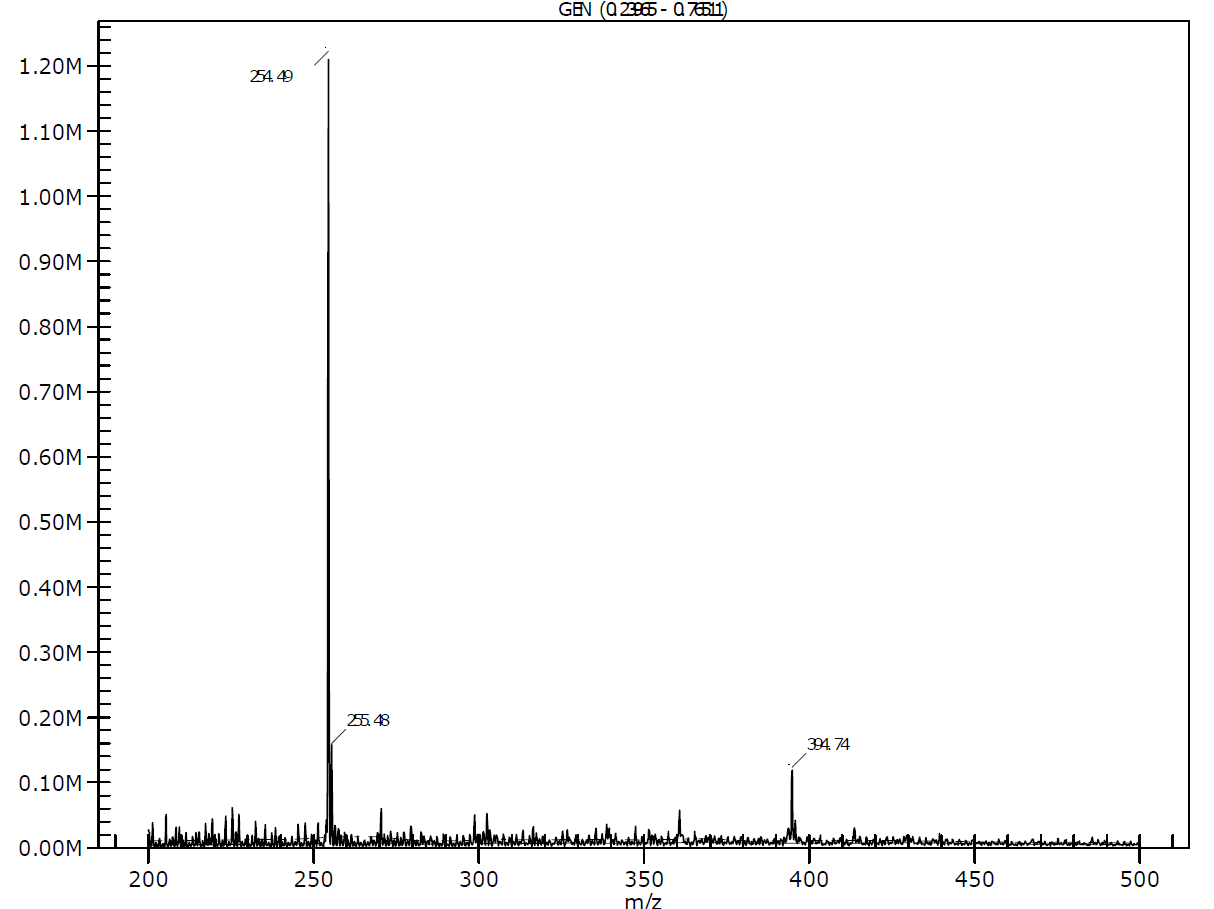


Figure S2: ESI-MS spectra of FucMAm


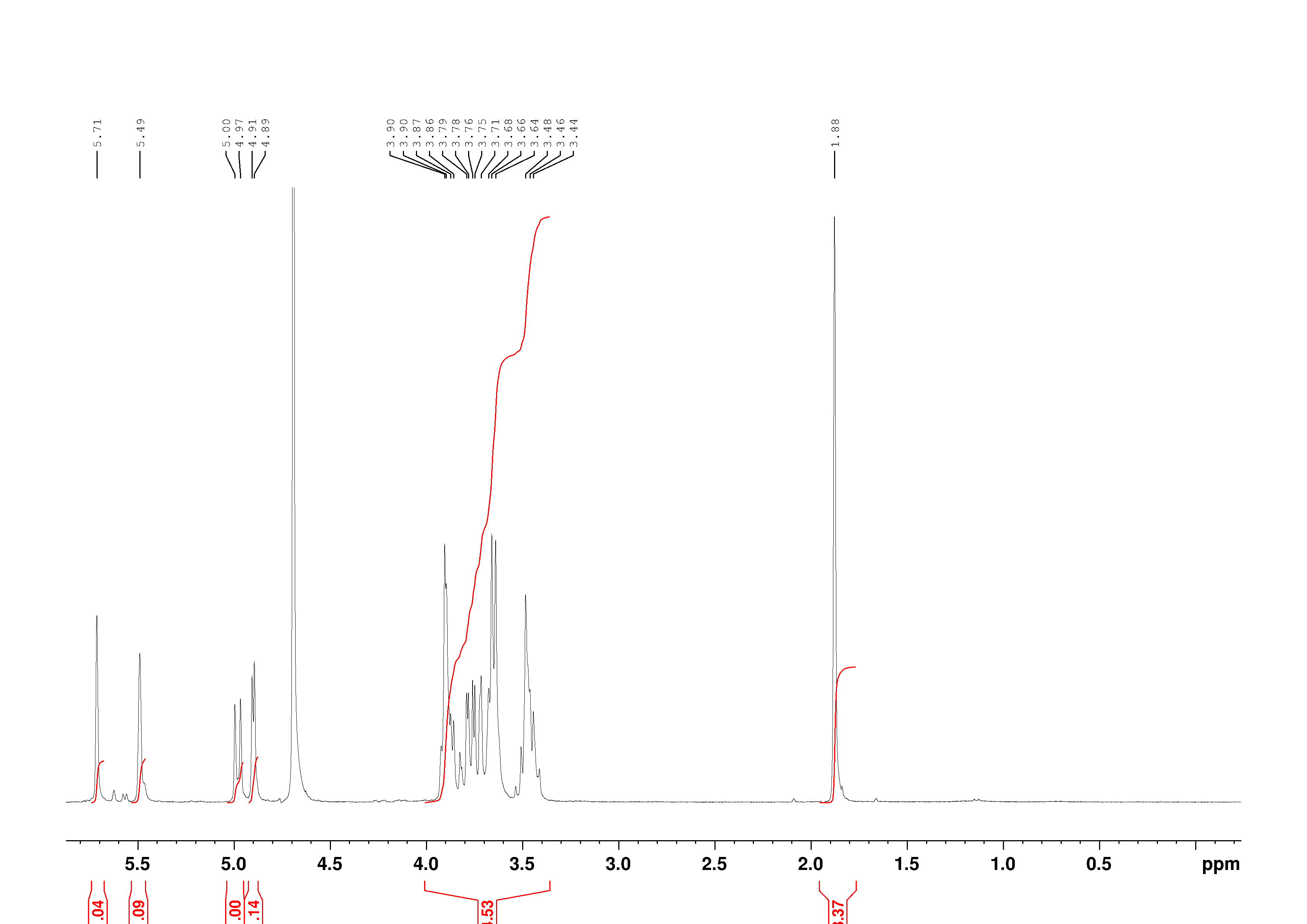


Figure S3: 1H-NMR spectra of MelMAm


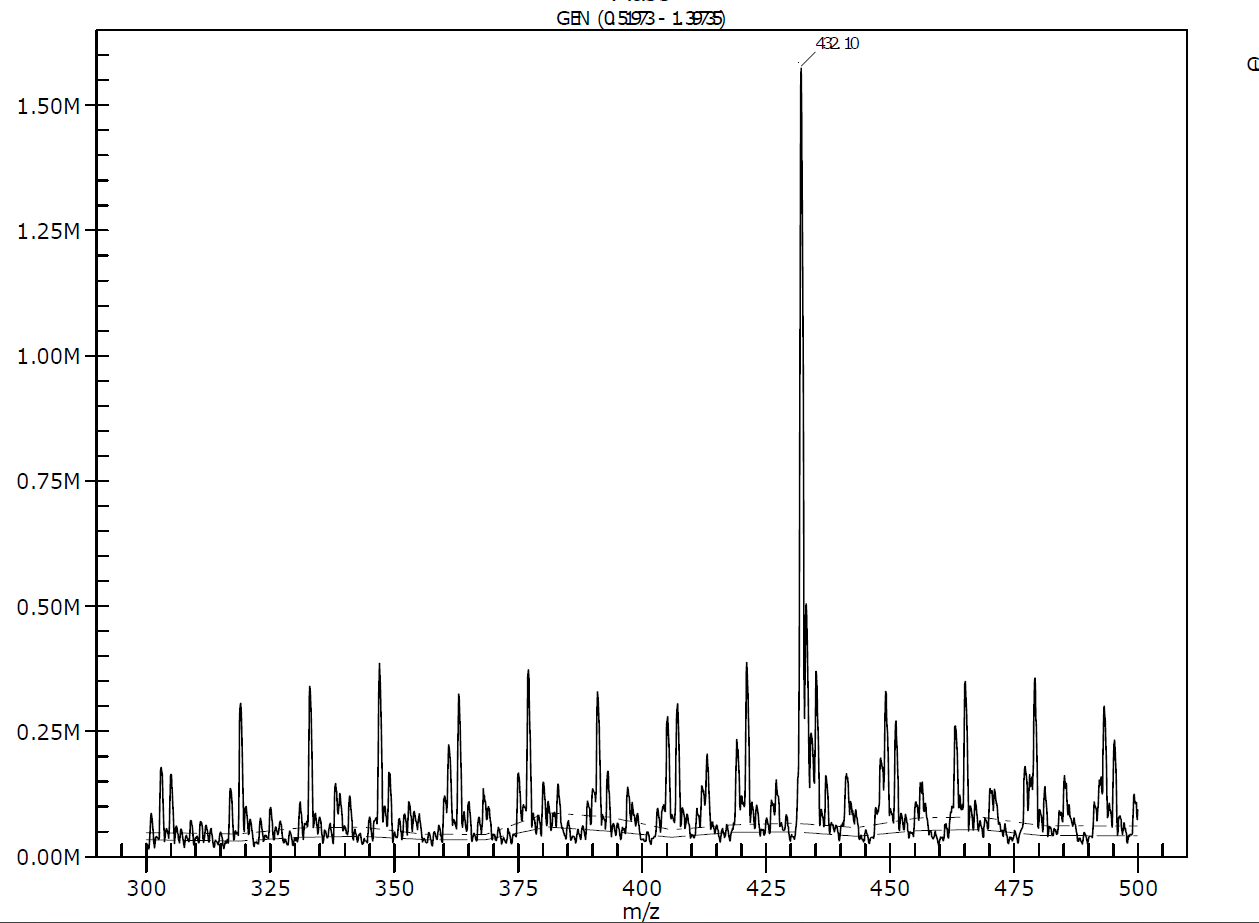


Figure S4: ESI-MS spectra of MelMAm


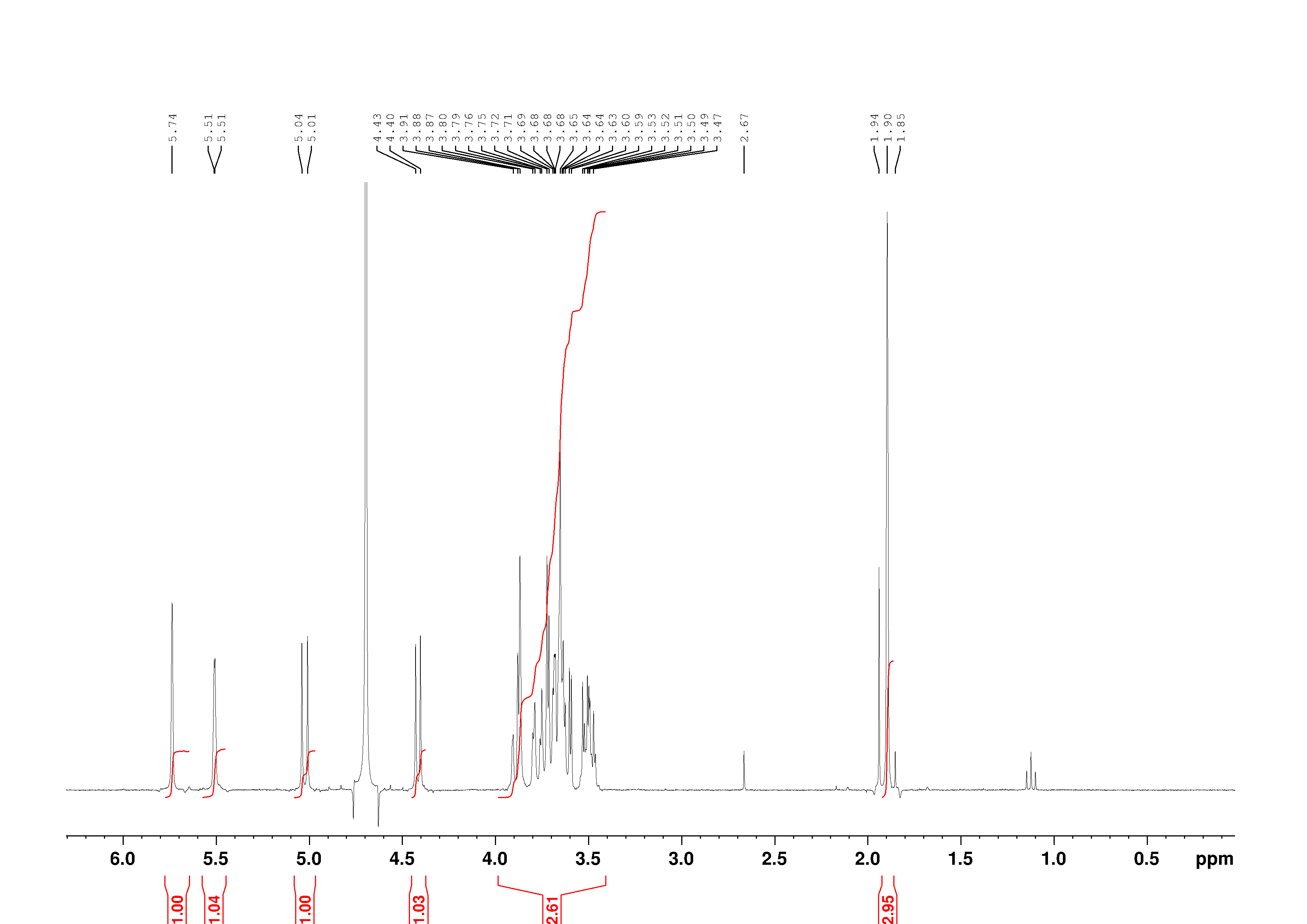


Figure S5: 1H-NMR spectra of LacMAm


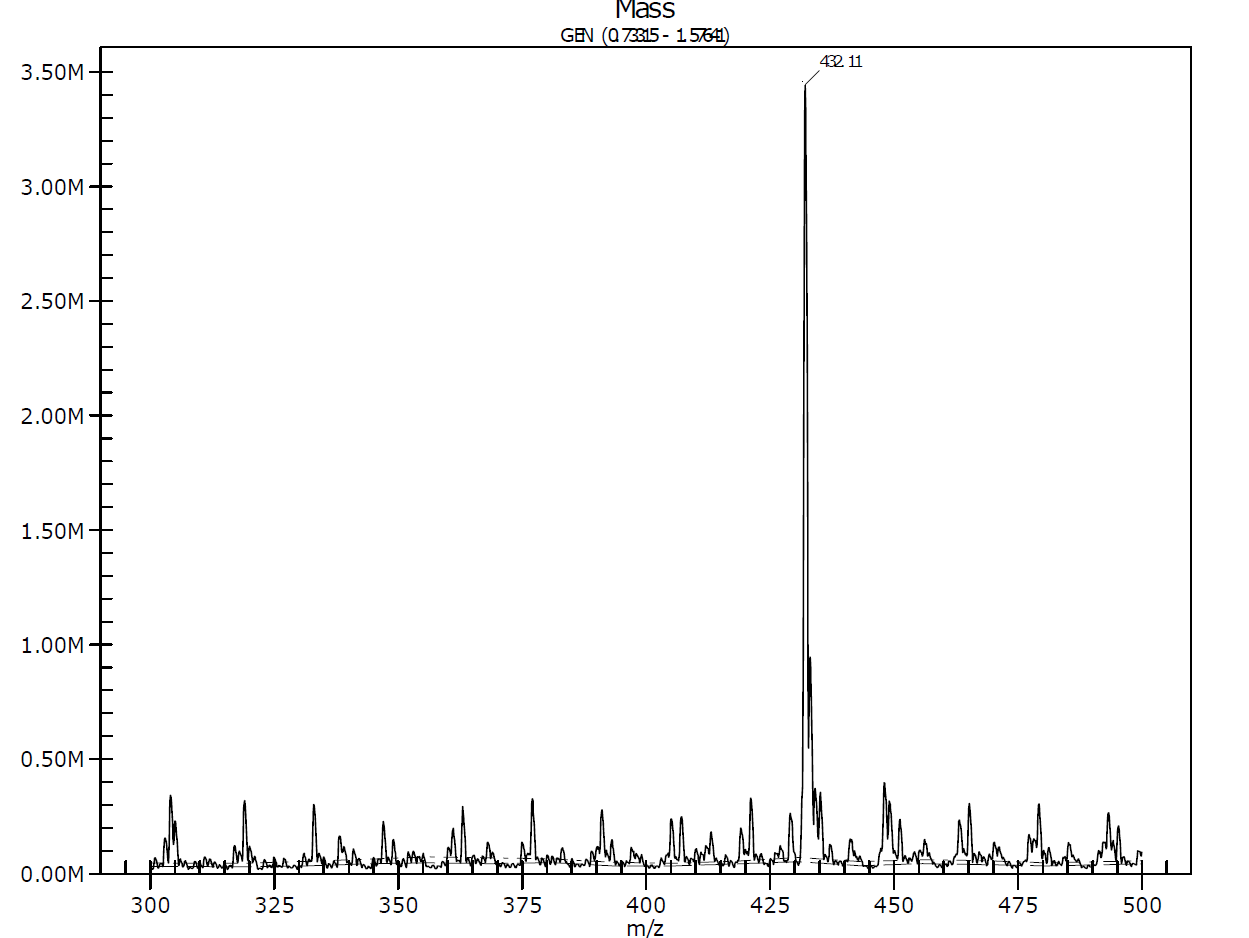


Figure S6: ESI-MS spectra of LacMAm
